# Contribution and Effectiveness of Laboratory Testing in the Diagnostic Assessment of Juvenile Ischemic Stroke and Transient Ischemic Attack

**DOI:** 10.7759/cureus.29256

**Published:** 2022-09-17

**Authors:** Francesco Janes, Roberta Giacomello, Francesco Blarasin, Martina Fabris, Simone Lorenzut, Gian Luigi Gigli, Francesco Curcio, Mariarosaria Valente

**Affiliations:** 1 Department of Neurosciences, Azienda Sanitaria Universitaria Friuli Centrale Santa Maria della Misericordia, udine, ITA; 2 Department of Medical Area, University of Udine, Udine, ITA; 3 Department of Microbiology, Azienda Sanitaria Friuli Occidentale Santa Maria degli Angeli, Pordenone, ITA; 4 Department of Laboratory Medicine, Azienda Sanitaria Universitaria Friuli Centrale Santa Maria della Misericordia, Udine, ITA; 5 Department of Neurosciences, Azienda Sanitaria Universitaria Friuli Centrale Santa Maria della Misericordia, Udine, ITA

**Keywords:** clinical laboratory quality management, ischemic cerebrovascular disease, laboratory finding, serum biomarkers, stroke protocol, young onset stroke

## Abstract

Introduction

Strokes in young people require an extensive diagnostic workup to detect their possible several etiopathogenetic mechanisms. There is no consensus indicating what and when it should be tested. The clinical benefit and cost-effectiveness ratio of laboratory tests is unclear as well.

Methods

In one series of 104 consecutive juvenile ischemic stroke patients, under 45 years old, admitted between January 1, 2012, and December 31, 2017, we considered a wide panel of laboratory biomarkers exploring both the patient’s basal status and specific risk factors for thrombotic disorders. To combine conventional and unconventional risk factors, structural defects, and other stroke-related diseases, we defined four categories of etiologic probability. We then studied the contribution of laboratory testing in changing the rate of “definite or probable stroke etiology” and the “proportion of patients with at least one additional risk factor” for stroke.

Results

The mere clinical assessment clarified stroke etiopathogenesis in 31% of cases. Abnormal values of the panel of biomarkers we considered were found in 30.1% of young ischemic strokes, while 11.5% of patients had unclear or borderline values. The benefit of laboratory assessment consisted of a relevant 14% gain in patients with a “definite or probable stroke etiology.”

Conclusion

Several areas of uncertainty are still pending and herein discussed, such as the low re-testing rate during follow-up and the neglect of some relevant biomarkers. However, our results support the importance of laboratory testing in this setting. An improvement of diagnostic protocols in juvenile ischemic stroke would even increase their effectiveness, and this is still an unsolved issue in the field of cerebrovascular diseases. The same age limit, conventionally considered for juvenile stroke, could be better defined according to the effectiveness of both laboratory and clinical assessment in identifying unconventional stroke risk factors.

## Introduction

Stroke is a constantly growing disease on a global scale, being the second leading cause of death and the third leading cause of global disability [1]. The prevalence of stroke increases with age, especially over 45 years [2], but juvenile stroke represents a peculiar, though minor, subset of patients. In fact, it can be caused by several different etiopathogenesis, requiring an extensive diagnostic workup. Its physical consequences are borne for decades, and the preventive therapeutic choices need to consider their impact on working activities and childbearing age. In juvenile stroke, the demand for laboratory tests - aimed to confirm specific etiopathogenesis and to detect non-classical or unconventional risk factors - is very high, often with unclear clinical benefit and cost-effectiveness.

In the neurological community, there is no consensus nor a solid guideline about what should be suggested to test and when it would be better to test it. This study aims at evaluating the actual clinical contribution of laboratory testing in the event of juvenile stroke by exploring the rate of change in patients with a “definite” or “probable” stroke etiology and by identifying at least one additional risk factor for stroke in an individual patient. The results of this study might prove helpful to discuss, organize, and establish protocols for laboratory testing in juvenile stroke.

## Materials and methods

Patients’ selection

We retrospectively collected data on consecutive stroke patients, between 0 and 45 years old, admitted to our tertiary care Hospital, from January 1, 2012, to December 31, 2017. The age limit we applied to define juvenile stroke was the most conservative one from the clinical point of view, according to previous studies on the subject [2-9].

The crude dataset of patients was obtained from Hospital Discharge Records, based on ICD-9-CM (International Classification of Diseases, Ninth Revision, Clinical Modification) classification [10], after selection of codes related to cerebrovascular diseases (codes 430-437).

The study was considered exempt from formal Ethical Committee approval because of its retrospective and data reviewing nature. Data were managed according to the Helsinki of Declaration principles, in an anonymous and confidential fashion, protecting the patients’ privacy.

We found a total of 175 records from Neurology and Pediatric Departments; 10 of them were removed because of duplicated data, 3 because of incomplete clinical data, and 26 due to diagnosis other than cerebrovascular event (6 with migraine, 5 with conversion disorder, 3 with seizure, 2 with subdural hematoma, 2 with vertigo, 2 with lumbar radiculopathies, 1 with multiple sclerosis, 1 with traumatic brain injury, 1 with reversible cerebral vasoconstriction syndrome, 1 with encephalitis, 1 with alcohol abuse, 1 undetermined). After revision of the dataset, 136 patients were suitable for the final analysis.

Stroke subtypes definitions

Discharge diagnosis and clinical records were reviewed by a senior neurologist, and patients were classified into six subtypes of cerebrovascular events: ischemic stroke (IS), transient ischemic attack (TIA), intracerebral hemorrhage (ICH), subarachnoid hemorrhage (SAH), cerebral venous thrombosis (CVT), and other undetermined (UND) strokes. Diagnoses were attributed according to WHO definitions [11]. All patients selected for the analysis had brain imaging. For three patients with an acute cerebrovascular event, data in our Institutional Repository were lacking and were then classified as UND.

Risk factors and conditions related to stroke etiology 

In the analysis of data, the following conventional and unconventional vascular risk factors were considered: hypertension, dyslipidemia, smoke habit, diabetes, obesity, migraine, and hormone therapy. In addition, information on familiar history of vascular disease was collected, although they were not intended as risk factors in data analysis.

Structural defects, traumatic injuries, and concurrent diseases, highly or possibly related to stroke occurrence, were labelled as “conditions related to stroke etiology.”

Among cardiomyopathies, a particular attention was dedicated to the subcategory of PFO (patent foramen ovale) and its specificities: atrial septum aneurysm, hypermobile inter-atrial septum, prominent Eustachian valve, Chiari’s network, large vs. mild right-to-left shunt, and long tunnel. PFO was considered at “high risk” if greater than two of these characteristics were present, and “low risk” otherwise [12].

Carotid stenosis was considered among the “conditions related to stroke etiology” because it is highly correlated to other vascular risk factors already listed.

Because of its features in angiography, we chose to consider probable or definite CNS (central nervous system) vasculitis among the “conditions” category, and also if laboratory supporting data were lacking.

Biomarkers

The biomarkers considered are categorized into two distinct groups: the first group assessing the patients’ basal status (including complete blood count, first level hemostasis, and metabolic profile) and the second group aiming to further evaluate the possible causes of the stroke event. To reduce the influence of acute phase on the laboratory results, all the data considered in this analysis were selected from those obtained after a conventional period of stabilization of the vascular event (7-14 days after event). Below, we report the detailed list of the tests considered.

Tests exploring patients’ basal status are as follows:

i. Hematologic tests: complete blood count (CBC)

ii. First-level hemostasis tests: prothrombin time (PT), activated partial thromboplastin time (aPTT), fibrinogen (QFA), D-dimer (DD), antithrombin (AT)

iii. Lipid profile: triglycerides (Tg), high-density lipoprotein (HDL), low-density lipoprotein (LDL)

iv. Liver function tests: aspartate aminotransferases (AST), alanine aminotransferases (ALT), gamma-glutamyl transpeptidase (GGT), total bilirubin (TBIL)

v. Markers of inflammation: C-reactive protein (CRP).

Tests oriented to discover stroke causes are as follows:

i. Second-level hemostasis: C protein (CP), S protein (SP), lupus anticoagulant (LA), activated protein C resistance (APCR)

ii. Genetic tests: MTHFR polymorphisms (MTHFR C677T and A1298C), factor II G20210A (FII), factor V Leiden (FVL), factor V H1299R (FVH1299R)

iii. Metabolic risk factor: homocysteine (Hcy), vitamin B12 and Folate; lipoprotein (a) (Lp(a))

iv. Autoantibodies: ANA (anti-nuclear antibodies), ENA (extractable nuclear antigen antibodies), ANCA (anti-neutrophil cytoplasmic antibodies), anticardiolipin abs, antiB2GPI, RF (rheumatoid factor).

Patients underwent other specific tests during clinical follow-up to detect rare causes of stroke, both sporadic and familiar, according to clinical picture and history. However, we did not consider those tests in this analysis because they are rarely requested and are consequently of poor relevance in creating a preliminary decision workflow.

Operative definitions of "contributory causes of stroke"

To allow interpretation of clinical features (both conditions and risk factors), laboratory data, and their mutual relationship, we defined the following four categories of etiologic probability: (1) structural causes/conditions highly related to stroke etiology, (2) ≥2 vascular risk factors/conditions probably explaining stroke etiology, (3) UND stroke etiology with 1 risk factor or condition, and (4) UND stroke etiology without known risk factors or conditions. Within the specific aim of this study, we considered this classification more informative than the traditional TOAST, which was conceived to describe mainly strokes in older cohorts. In fact, two major TOAST categories, "large artery atherosclerosis" and "small vessel disease," are rarely represented in juvenile stroke, and other two, "cardio embolism" and "other determined etiologies," do not depict and detail the complex relationship between laboratory tests results and specific structural conditions underlining stroke.

We also pinpointed two operative definitions of the specific “contribution of laboratory data” to stroke etiologic categories: (1) the percentage increase in the proportion of patients allocated in category number 1-2 (i.e., the reduction in UND cases after lab assessment) and (2) the percentage increase in adding at least one risk factor to stroke assessment.

Reference intervals

Table 1 summarizes the reference intervals and cut-offs considered by our laboratory for different analytics. In the other sections of this study, we refer to abnormal values of a biomarker according to these intervals unless otherwise specified.

**Table 1:** Physiologic reference values for each biomarker considered Hct, hematocrit; Hb, hemoglobin; Plt, platelets; WBC, white blood cells; aPTT, activated prothrombin time; PT, prothrombin time; DD, D-dimer; QFA, quantitative fibrinogen; AT, antithrombin III; Hcy, homocysteine; CP, C protein; SP; S protein; APCR, activated protein C resistance; Tg, triglycerides; HDL, high-density lipoprotein; LDL, low-density lipoprotein; CRP, C-reactive protein; GGT, gamma glutamyl transferase; tBil, total bilirubin; AST, aspartate aminotransferase; ALT, alanine aminotransferase; Lp(a), a-lipoprotein; LAC, lupus anticoagulant; DRVVT, dilute Russell viper venom time; ANA, anti-nucleocytoplasmic antibodies; ENA, extractable nuclear antigen antibodies (specificities: Ro52, Ro60, Sm, U1-RNP, Scl-70, Jo-1, SSB); ANCA, anti-neutrophil cytoplasmic antibodies; anti-CL, anticardiolipin antibodies; RF, rheumatoid factor; C3 and C4, complement C3 and C4 levels *Further reference values were applied according to patients’ age and sex ♂ and ♀indicated male and female sex, respectively

	Reference		Reference		Reference		Reference
Hct (♂)	40.0–50.0 %	Hb (♂)	14–18 g/dL	Hct (♀)	37.0–50.0 %	Hb (♀)	12–16 g/dL
Plt	150–400 x 10^3^/μL	WBC	4.0–11.0 x 10^3^/μL	aPTT	0.80–1.20 ratio	PT	0.85–1.15 ratio
DD	< 500 FEU/ng/mL	QFA	180–380 mg/dL	AT	80–120%	Hcy	6–12 μmol/L*
CP	70–140 %	SP	58–155 %	APCR	< 2.2 ratio	Tg	40–150 mg/dL
HDL	> 35 mg/dL	LDL	< 125 mg/dL	CRP	0–5 mg/L	GGT	4–40 UI/L
tBil	0.2–1.00 mg/dL	AST	4–40 UI/L	ALT	4–41 UI/L	Lp(a)	0–20 mg/dL
C3	90–220 mg/dL	C4	10–40 mg/dL	Anti-CL	IgG 0–10 UI/mL; IgM 0–20 UI/mL	Anti B2GPI	IgG 0–8 UI/mL; IgM 0–20 UI/mL
LAC	DRVVT ≥ 1.2, Silica clotting time > 1.23	ANA, ENA, ANCA	< 1:160 to < 1:40(IFI)	antiDS-DNA	< 20; CLIFT +	FR	< 10

Statistical analysis and cost estimation

Continuous normally distributed variables were expressed as mean ± SD, otherwise as median of the interquartile range (IQR). Dichotomic variables were expressed as number and percentage. The chi-square test and Student’s t-test were used for categorical and continuous variables, respectively. If the continuous variable was not normally distributed, the Mann-Whitney U test was alternatively used. A p-value of ≤0.05 was considered statistically significant.

After referring to the price list published by our institution [13] to assign cost to each biomarker that had been tested, the “overall cost” of laboratory analysis and a “mean cost per patient” were calculated, with the latter based on the comprehensive cost of all biomarkers analyzed per single patient.

## Results

**Figure 1:**
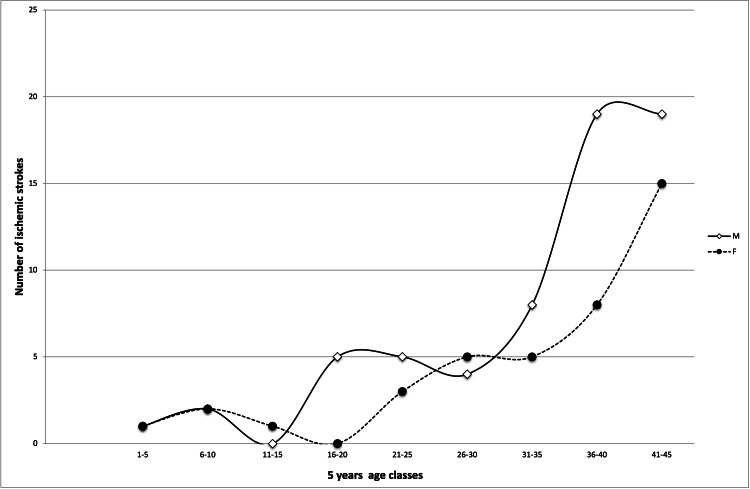
Age and gender distribution of ischemic stroke patients

A total of 136 patients were included in the dataset, with 104 (76.5%) of them with an ischemic type of acute vascular event, 84 (61.8%) with IS, and 20 (14.7%) with TIAs. Overall, 77 patients were male (56.6%), while female patients were 59 (43.4%). Figure 1 shows detailed patients' age and sex distribution. In total, 25 patients had an hemorrhagic stroke, 18 (13%) had ICH, 7 (5%) had SAH, 3 (2%), had a deep vein thrombosis, and 3 (2%) an UND stroke. Overall, 125 (91.9%) patients were of Caucasian ethnicity (113 western European, 12 eastern European), 3.7% were African, 2.9% were Hispanic, and 1.5% were Middle Eastern.

The following results refer to ischemic types of cerebrovascular accidents (IS and TIA) only. The baseline characteristics, clinical vascular risk factors, and comorbidities of our case series are summarized in Table 2.

**Table 2:** Baseline features, vascular risk factors, and conditions in ischemic cerebrovascular accidents TIA, transient ischemic attack; IS, ischemic stroke; RRFF, risk factors; PFO, patent foramen ovale; IAD, interatrial defect; ASA, atrial septum aneurysm; ROPEs, risk of paradoxical embolism score; CNS, central nervous system; VZV, Varicella zoster virus; HSV, herpes simplex virus

	TIAs (N = 20), n (%) or n (±SD)	ISs (N = 84), n (%) or n (±SD)	All ischemic events (N = 104), n (%) or n (±SD)
Age	35.25 (±7.63)	33.72 (±11.01)	34.02 (±10.42)
Sex (female)	5 (25.0)	37 (44.0)	42 (40.4)
Ethnicity	18 (90.0) Caucasian	69 (82.1) Caucasian	87 (83.6) Caucasian
	1 (5.0) Eastern European	9 (10.7) Eastern European	10 (9.6) Eastern European
	1 (5.0) Hispanic	3 (3.6) Hispanic	4 (3.8) Hispanic
	0 (0.0) African	3 (3.6) African	3 (2.9) African
	0 (0.0) Asian	2 (2.4) Asian	2 (1.9) Asian
Conventional RRFFs			
Smoke	7 (35.0)	27 (32.1); Past smokers = 2 (2.4)	34 (32.7)
Hypertension	5 (25.0)	15 (17.9)	20 (19.2)
Dyslipidemia	3 (15.0)	11 (13.1)	14 (13.5)
Obesity	1 (5.0)	4 (4.8)	5 (4.8)
Diabetes	1 (5.0)	3 (3.6)	4 (3.8)
Unconventional RRFFs			
Migraine	Overall = 0 (0)	12 (14.3)	12 (11.5)
	-	With aura = 6/12	-
	-	Without aura = 6/12	-
Hormone therapy	0 (0)	4 (4.8)	4 (3.8)
Pathological conditions/structural vascular defects			
PFO	7 (35.0)	40 (47.6)	47 (45.2)
	High-risk PFO = 4/7 (57.1)	High-risk PFO = 11/40 (27.5)	High-risk PFO = 15/47 31.9)
	ASA = 0/7 (0.0)	ASA/IAD = 6/40 (15.0)	ASA/IAD 6/47 (12.8)
	ROPEs = 6.71 (±0.76)	ROPEs = 7.94 (±1.33)	ROPEs = 7.74 (±1.33)
Other cardiomyopathies	2 (10.0)	7 (8.3)	9 (8.6)
	Low embolic risk = 2	Low embolic risk = 4	Low embolic risk = 6
	High embolic risk = 0	High embolic risk = 3	High embolic risk = 3
Artery dissection	0 (0)	5 (5.9)	5 (4.8)
	Traumatic dissection = 0	Traumatic dissection =2/5	Traumatic dissection =2/5
	Spontaneous dissection = 0	Spontaneous dissection = 3/5	Spontaneous dissection = 3/5
Structural artery disease	1 (5.0)	2 (2.4)	3 (2.9)
	1 carotid agenesis	1 brainstem/cervical MAV	-
	-	1 intracranial carotid a. stent	-
Carotid stenosis	1 (5.0)	9 (10.7)	10 (9.6)
Defined CNS vasculitis	2 (10.0)	2 (2.4)	4 (3.8)
Family history of vascular disease	0 (0)	5 (5.9)	5 (4.8)
Miscellaneous	0 (0)	12 (14.3)	12 (11.5)
		5 = concurrent infection (1 Lyme; 2 VZV; 1 HSV; 1 pneumonia)	
		2 = Crohn's disease	
		1 = Sotos’ syndrome	
		1 = Gaucher’s disease	
		1 = Whole brain radiotherapy	
		1 = Cocaine abuse	
		1 = Neurofibromatosis 1	
Number of RRFF/conditions			
0	3 (15.0)	7 (8.5)	10 (9.8)
1	8 (35.0)	38 (46.3)	46 (45.1)
2	6 (30.0)	26 (31.7)	32 (31.4)
≥3	3 (20.0)	11 (13.4)	14 (13.7)

**Figure 2:**
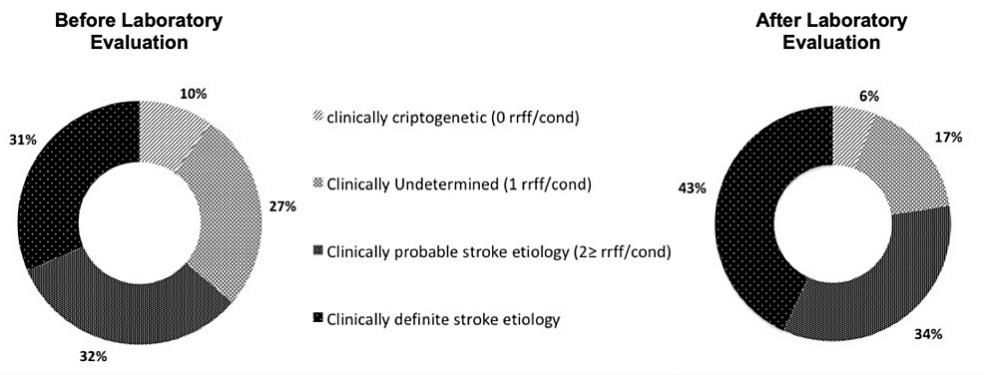
Proportion of the four classes of “contributory causes of stroke” before and after laboratory assessment

Figure 2 (pie chart labelled “before laboratory evaluation”) shows the proportion of the patients allocated into the four “clinical etiological probability classes.” The clinical assessment clarified the etiopathogenetic background in 32/104 (31%) ISs, mainly because of the evidence of structural defects of vessels (e.g., artery dissection, arteriovenous malformation, vasculitis) and of the heart (e.g., interatrial defect, myxoma, endocarditis, high-risk PFO) known to be directly involved in thrombus formation alone or in conjunction with other concurrent risk factors. Noteworthy, 8/32 (25%) of them were assigned to this category due to evidence of high-risk PFO only; 37% of patients were still cryptogenic after extensive clinical workup.

**Figure 3:**
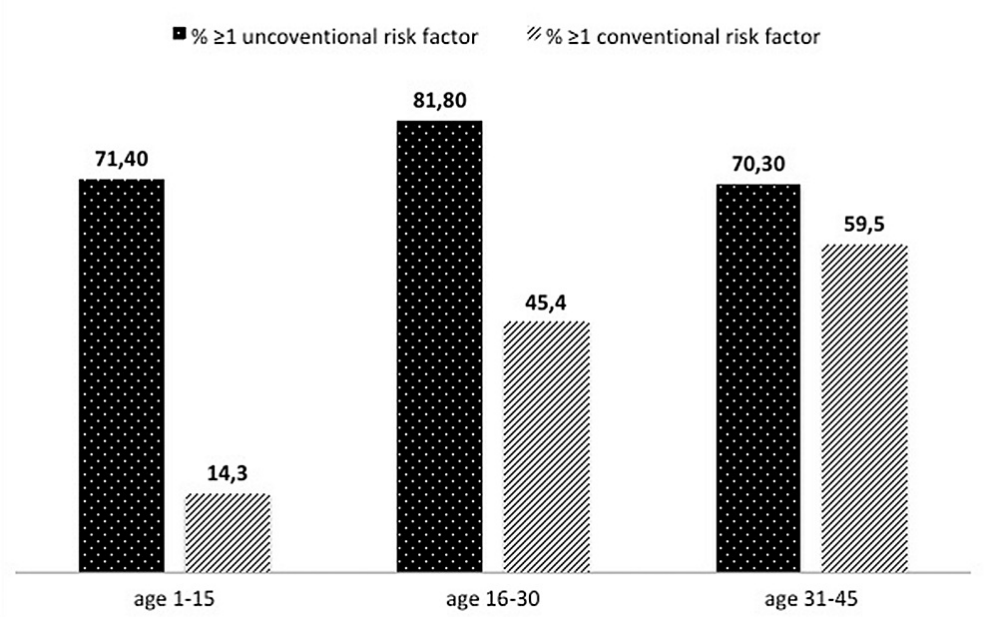
Age trend in the proportion of conventional and unconventional risk factors in juvenile ischemic strokes

Overall, 55/104 patients (53.4%) had at least one conventional risk factor, 14 (13.6%) had ≥ 2 risk factors, and 9 (8.7%) ≥ 3 risk factors. Figure 3 shows that conventional risk factors prevalence clearly increased with patient aging.

**Figure 4:**
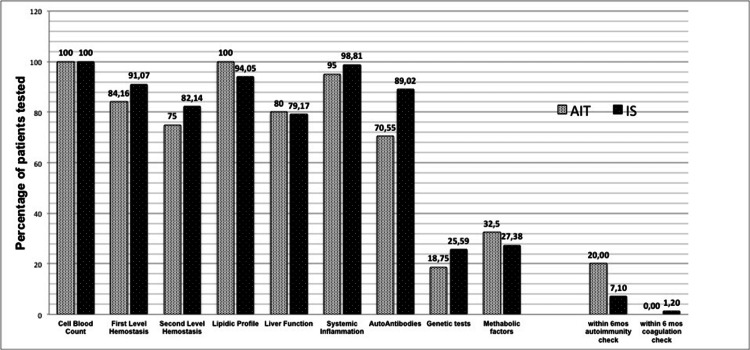
Proportion of ischemic patients undergoing laboratory examination This figure presents the categories of laboratory exams (see Table 1 for a complete list and reference values of each analytic); the rate of repetition within six months after stroke is reported on the right.

Figure 4 summarizes the proportion of patients who underwent specific categories of laboratory tests. Noteworthy, 10 patients (9.6%) only repeated those exams within six months from stroke event (nine repeated “systemic autoantibodies” only and one “second level hemostasis” only).

Laboratory workup returned 30.1% of abnormal results (32/104 patients with at least one additional laboratory risk factor). A further 11.5% had unclear results, non-specific or borderline. Noteworthy is that none of the 12 patients who had high-risk vascular laboratory findings had a concurrent “clinically definite stroke etiology.” Laboratory results are summarized in Tables 3, 4. Few patients underwent laboratory tests other than those here reported: 10 patients underwent a-GAL (alpha-galactosidase) activity screening and they all showed normal activity except for one (second-level GLA genetic test was negative in this last case), two patients were tested for an illicit drug panel in the ER with negative results, and one patient proved negative for Ehlers-Danlos’ disease.

**Table 3:** Occurrence of laboratory pathologic findings in ischemic stroke types (N = 104) PL, phospholipid; LAC, lupus anticoagulant; Lp(a), a-lipoprotein; aCL abs, antiCardioLipin antibodies

	Vascular risk factor	Number of patients – laboratory and clinical details
High-risk factors/conditions	Dysimmune disorders	3 – Undifferentiated connective tissue disease
	Anti-PL syndrome	3 – LAC+
		1 – Triple positive
	Genetic factors	1 – Homozygous C677T-MTHFR
		1 – Homozygous A1298C-MTHFR
		1 – Homozygous F-V Leiden
		1 – Double heterozygous A1298C MTHFR + G20210A-FII
	Synergic risk factors	1 – Concurrent increased LP-a, polycythemia, and hyperhomocysteine
Isolated factors	Unknown dyslipidemia	10
	Increased LP-a	6
	Polycythemia	3
	Genetic factors	3 – Heterozygous F-V Leiden
		1 – Heterozygous C677T-MTHFR
		1 – Heterozygous G20210A - F-II
	Moderate hyperhomocysteine	1
	Thalassemic trait	1
Non-definite factors	Decreased S protein	2
	Decreased P protein	1
	Low-titer autoantibodies	4
	Mild hyperhomocysteinemia	1
	Isolated increased D-dimer	4
	aCL abs +/ LAC -	1

**Table 4:** Summary of results of laboratory analysis in ischemic strokes Hct, hematocrit; Hb, hemoglobin; Plt, platelets; WBC, white blood cells; aPTT, activated prothrombin time; PT, prothrombin time; DD, D-dimer; QFA, Quantitative fibrinogen; AT, antithrombin III; Hcy, homocysteine; CP, C protein; SP; S protein; APCR, activated protein C resistance; Tg, triglycerides; HDL, high-density lipoprotein; LDL, low-density lipoprotein; CRP, C-reactive protein; GGT, gamma glutamyl transferase; tBil, total bilirubin; AST, aspartate aminotransferase; ALT, alanine aminotransferase; Lp(a), a-lipoprotein; LAC, lupus anticoagulant; ANA, anti-nucleocytoplasmic antibodies; ENA, extractable nuclear antigen antibodies (specificities: Ro52, Ro60, Sm, U1-RNP, Scl-70, Jo-1, SSB); ANCA, anti-neutrophil cytoplasmic antibodies; RF, rheumatoid factor; C3 and C4, complement C3 and C4 levels

	AIT (N = 20), mean (standard deviation) or N positive / N of analyses	IS (N = 84), mean (standard deviation) or N positive / N of analyses
Hct (%)	42.54% (0.04)	41.82% (0.05)
Hb (g/dL)	14.2 (1.18)	13.74 (1.75)
Plt (x10^3^/μL)	188.3 (30.2)	238.2 (76.5)
WBC (x10^3^/μL)	7,200.00 (2,140.00)	8,412.8 (3,072)
aPTT (ratio)	1.01 (0.10)	1.02 (0.14)
PT-INR	1.02 (0.07)	1.05 (0.10)
DD (FEU ng/mL)	395.42(520.31)	881.09 (2,500.65)
QFA (mg/dL)	313.50 (60.64)	311.91 (86.26)
AT III (%)	101.21 (10.85)	102.11 (14.13)
Hcy (μm/L)	11.50 (2.09)	13.07 (6.89)
PC (%)	103.56 (21.61)	106.93 (22.95)
PS (%)	90.81 (22.21)	91.11 (19.77)
APCR	2.93 (0.18)	2.86 (0.37)
Tg (mg/dL)	124.70 (74.54)	111.21 (80.99)
HDL (mg/dL)	47.20 (10.01)	54.67 (18.50)
LDL (mg/dL)	103.60 (30.05)	110.27 (44.84)
CRP (mg/L)	1.21 (2.08)	5.45 (12.69)
GGT (UI/L)	24.44 (20.11)	27.96 (32.95)
tBIL (mg/dL)	0.94 (0.78)	0,78 (1.25)
AST (UI/L)	19.70 (7.36)	18.87 (8.78)
ALT (UI/L)	21.35 (11.91)	21.99 (19.72)
LP(a) (mg/dL)	13.33 (16.81)	19.91 (19.02)
LAC	0/16	5/77
Anti-cardiolipin Abs	0/15	3/76
Anti-β2GpI Abs	0/15	2/76
ANA	Negative – 12/14	Negative – 60/76
	1:80 – 0/14	1:80 – 5/76
	1:160 – 1/14	1:160 – 9/76
	≥1:320 – 1/14	≥1:320 – 2/76
ENA	Negative – 13/14	Negative – 75/76
	SSA/Ro+SSB/La positive – 1/14	SSA/Ro positive – 1/76
ANCA	Negative – 13/13	Negative – 66/74
	-	1:80 – 2/74
	-	1:160 – 3/74
	-	≥1:320 – 3/74 (MPO+/PR3+ - 0/3)
RF (UI/mL)	<10 – 13/14	<10 – 73/74
	>10 – 1/14	>10 – 1/74
C3 (mg/dL)	112.08 (25.12)	109.38 (19.65)
C4 (mg/dL)	23.43 (8.46)	24.3 (5.97)
AntiDS-DNA (UI/mL)	<20 – 14/14	<20 – 71/75
	>20 – 0/14	>20 – 4/75 (clift+ - 0/4)

Figure 2 (pie chart labelled “after laboratory evaluation”) shows the proportion of the four classes of stroke etiology, as it has been modified after the contribution of laboratory testing. Compared to stroke etiology before laboratory evaluation, the net benefit of laboratory testing consists of a 14% gain in patients allocated to a “probable/definite stroke etiology.”

The cost of the laboratory assessment that we considered was € 260.00 per patient (SD = 109.20), and € 27,818.00 for the total series.

## Discussion

Although almost 40% of juvenile strokes is reported to remain cryptogenic, even after extensive clinical and instrumental workup [14], little is known about the usefulness and the effectiveness of laboratory assessment in juvenile stroke. Guidelines substantially lack in indications about which tests should be requested and the timing of the tests in relation to stroke onset. The main issue seems the difficulty to achieve a reliable operative standard because of the interplay among the deep etiological heterogeneity of juvenile stroke, the variable prevalence of genetic and metabolic factors in different populations, and the relatively small incidence of juvenile stroke. To clearly define the boundaries among risk factors, structural conditions, and triggers of stroke seems critical to both understand stroke etiology and establish diagnostic indications. Additionally, a recent study by Aigner et al. [15] documented that classical modifiable risk factors for stroke are frequently reported in young patients, consequently suggesting that a detailed and complete clinical assessment is another baseline key element to judge the effectiveness of laboratory tests themselves. The age of the 87.2% of Aigner’s ISs sample ranged between 35 and 55 years. Differently, we applied a strict age limit of <45 years old with the aim of detecting juvenile strokes highly related to unconventional causes. Despite the lower upper age limit, the frequency of conventional risk factors over 35 years is high up to 60% in our sample as well (Figure 4), even if we did not consider physical activity and alcohol consumption (data unfortunately not available in most of our records). However, from our data, unconventional factors seem still very frequent, possibly more than conventional ones, in the age range of 31-45 years: this finding justifies in our opinion the need for a more extensive clinical-laboratory assessment in patients at least up to 45 years old. Indeed, the conventional age limit between young and old varies in the literature, mainly from 45 to 55 years [14-17], but as high as 60 or 65 years in some studies [18-19]. Our results suggest that, in the future, after further analysis extended up to 65 years old, this limit might be operatively defined once forever by the age in which traditional vascular factors would start to overcome unconventional structural and laboratory factors.

The information emerging from the few previous studies on this topic [18-21] are controversial; the results of laboratory tests in case series are different, as well as the panel of test used. In some studies, the relevance (e.g., their importance in changing therapeutic approach) of the additional unconventional risk factors seems to be very low. Our experience adds more data, both clinical and laboratory, but also aims at creating a more critical point of view on this topic. In fact, the complexity of juvenile stroke cannot neglect the need for a long-lasting vascular prevention therapy and/or immune-modulating therapy; the effectiveness of preventive therapies on stroke recurrence and the patients’ adherence to long-term prescriptions are not well known in the context of unconventional risk factors and young people, respectively.

 We observed a high rate of generalization (i.e., more than 75% of patient sample) in basal testing, first- and second-level autoimmunity, and first- and second-level hemostasis. On the contrary, we found a low rate of request of genetic testing and metabolic risk factors. “Basal status markers” refer to a very common and first-level laboratory assessment of acute stroke phase, but it is poorly informative on stroke etiology. Second-level immunological, thrombophilic, metabolic, and genetic tests are otherwise more oriented to detect stroke etiology. In our dataset, approximately 80% of patients underwent second-level testing.

The markers reported here are almost all listed in the stroke laboratory assessment of most previous studies, but other potential markers of vascular pathology, although well-known causal factors of ischemia, are rarely part of the panel of juvenile stroke laboratory assessment. The following are a few examples of such potential markers belonging to completely different categories and pathologies: illicit drugs screening [22], LP(a) levels [23], Von Willebrand factor, ADAMTS (a disintegrin and metalloproteinase with thrombospondin motifs), and antibodies against ADAMTS [24]. They might reveal new insights into the pathogenesis of stroke, but these and other markers are almost only reported in specific literature, often not directly addressed to stroke specialists.

One of the key issues highlighted by this study was the limited follow-up rate of laboratory testing, considering a six months’ period from vascular event. This is probably responsible of a diminished effectiveness of laboratory testing in defining stroke etiopathogenesis. Alakbarzade et al. observed the same trend toward a poor follow-up of pathologic or borderline findings in their series [19]. They also found a 14% of abnormal findings and stated that this low impact of laboratory testing did not seem to justify a generalized workup in young stroke patients. They further remarked that the policy of requesting laboratory detailed examination should be somehow individualized and should at least focus on patients without conventional or structural emerging factors. Interestingly, in our dataset, all the laboratory risk factors important for diagnostic decision emerged in patients without conventional risk factors or conditions that explained stroke etiopathogenesis, partially corroborating Alakbarzade et al.’s observations. However, in our analysis, we included more and different markers, removed hemorrhagic and UND stroke types, and selected a lower age limit; these differences do not allow a clear comparison with our results. In any case, we think that a refinement of laboratory workup - based on increasing published data - is likely to significantly improve its effectiveness.

Another relevant point rising from this analysis is the lack in clear indications about when a specific test should be requested. Most of the hemostasis data are affected by acute phase effect and they should at least be repeated three months after stroke onset. On the contrary, genetic testing can be performed indifferently along the stroke course. Illicit drug screening must be performed in hyperacute phase of stroke. These three examples underline the need for a more detailed timing of laboratory testing. A multistep timing of laboratory testing might be built according to the pathophysiology of the specific marker. However, data herein presented cannot be conclusive for this purpose. The results of ongoing multicenter trials on this topic, such as the SECRETO study [25], will be of further help in paving the way to a comprehensive protocol of laboratory testing in stroke patients.

Our analysis has a few limitations. We hinted at some of them earlier in the discussion: the arbitrary age limit for juvenile stroke and the low rate of tests’ repetition during follow-up. We did not run subanalysis to explore gender and ethnicity diversities, basically due to the relatively small sample size of our series. For these and other reasons, multicenter studies would be undoubtfully desirable. The panel of biomarkers we chose, and their timing, are the most controversial issues in this field and hamper a clear generalization of our results.

## Conclusions

Which laboratory tests and when to test them in the setting of juvenile stroke is still a matter of research and debate. However, the results we showed herein suggest that a panel of widely available biomarkers, tested in the acute phase of IS, usefully lead to an increase of definite stroke etiologies. Although this contribution is clinically relevant, several ISs remain undetermined. Consequently, the improvement of diagnostic protocols should include new biomarkers, indicate when it would be better to test them, and which biomarkers should be retested during follow-up. To achieve higher rates of definite etiologies in young stroke will be crucial also to understand the boundary line between stroke in the young and in the elderly.
